# Loss of nuclear BAP1 expression is associated with poor prognosis in oral mucosal melanoma

**DOI:** 10.18632/oncotarget.16175

**Published:** 2017-03-14

**Authors:** Hao Song, Lizhen Wang, Jiong Lyu, Yunteng Wu, Wei Guo, Guoxin Ren

**Affiliations:** ^1^ Department of Oral & Maxillofacial-Head and Neck Oncology, Shanghai Ninth People's Hospital, Shanghai Jiaotong University, School of Medicine, Shanghai, 200011, China; ^2^ Department of Oral Pathology, Shanghai Ninth People's Hospital, Shanghai Jiaotong University, School of Medicine, Shanghai, 200011, China; ^3^ Department of Stomatology, Zhejiang University School of Medicine First Affiliated Hospital, Hangzhou, Zhejiang Province, 310003, China

**Keywords:** oral mucosa, melanoma, BAP1, prognosis, immunohistochemistry

## Abstract

Oral mucosal melanoma (OMM) is an aggressive neoplasm with an extremely poor prognosis. BAP1 is a tumor suppressor that has been associated with the outcome of melanomas and other malignancies. In this study, we investigated the genetic alterations in *BAP1* and the prognostic potential of BAP1 protein expression in oral mucosal melanoma. DNA sequence analysis of *BAP1* from 12 OMM patient samples revealed missense mutations in the tissues from four patients. Based on immunohistochemical staining, loss of nuclear BAP1 expression was associated with poor overall survival (*P* < 0.001, Log-rank = 21.308) and distant metastasis (*P* = 0.034, OR = 0.320). Multivariate analysis showed *BAP1* to be an independent prognostic factor (*P* = 0.027, HR = 0.479). It thus appears that loss of nuclear BAP1 expression is an independent prognostic factor of poor overall survival and associated with distant metastasis in OMM.

## INTRODUCTION

Oral mucosal melanoma (OMM) is an aggressive neoplasm accounting for nearly 1-8% of all melanomas arising in the oral mucosa and is characterized by a high rate of distant metastasis and extremely poor outcomes [1–3]. OMM differs from cutaneous melanoma in regard to etiology, histopathology, genetic alterations and prognosis [6, 7]. However, the pathogenesis and prognosis of OMM are poorly understood.

BAP1 is a deubiquitinating enzyme associated with multi-protein complexes that regulate cell cycle, cellular differentiation, cell death and DNA damage response that are key for carcinogenesis [8]. The *BAP1* gene locus is located on chromosome 3 (3p21.1) and frequent deletions of the 3p21 region has been commonly observed in lung and breast cancer cell lines [9]. In addition, germline and/or somatic mutations in *BAP1* are reported in uveal melanoma, atypical epithelioid Spitz tumors, cutaneous melanoma, mesothelioma, renal cell carcinoma, lung adenocarcinoma, meningioma and many other cancers [9]. BAP1 was initially reported to be associated with the BRCA1 RING finger domain [10]. However, later studies showed that although BAP1 was a tumor suppressor that played a role in BRCA1-mediated processes, it performed BRCA1-independent functions as well [9]. Inactivation mutations in *BAP1* have been associated with the pre-disposition and outcomes of many malignant tumors. Harbour and co-workers showed inactivating *BAP1* mutations in majority of metastasizing uveal melanomas [11]. Wiesner and colleagues demonstrated that BAP1 assessment by IHC (immunohistochemistry) was a useful tool for subtyping melanocytic neoplasms [12]. Koopmans and others found strong correlations between the BAP1 IHC and sequencing data in uveal melanoma [13]. Loss of *BAP1* was also associated with poor disease-free survival (DFS) and melanoma-specific survival (MSS) after adjusting for clinical and pathological factors in cutaneous melanoma [14]. Recently, loss of BAP1 expression has been used as a biomarker for therapeutic strategies [8, 15–17]. Our previous investigations regarding the treatment modalities and the prognosis of OMM revealed that the genetic alterations and the biomarkers of OMM remain to be elucidated [6, 7, 18–20]. Since BAP1 was closely associated with melanocytic tumors, we postulated that investigating its function may shed light on the pathogenesis and novel prognosis of OMM. Therefore, the aim of our study was to investigate the presence of *BAP1* mutations and the prognostic potential of BAP1 protein expression using OMM patient samples.

## RESULTS

### Sanger sequencing of BAP1 in OMM patient samples

**Figure 1:**
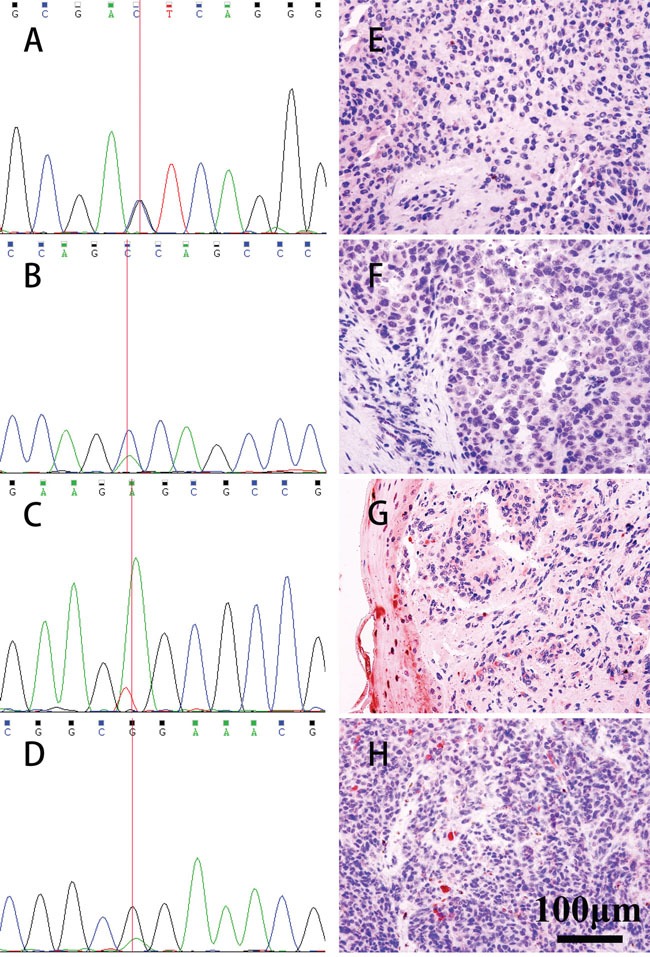
Mutations of *BAP1* exons detected by Sanger sequencing and analyzing the nuclear localization of the BAP1 protein by IHC Mutations were detected in 4 patients (**A**. Chr3: 52407995 C>G, **B**. p.S113T; Chr3: 52409864 C>A, **C**.p.W5C; Chr3: 52408565 T>A, p.E55V; **D**.Chr3: 52402325 C>T, p.R718Q). IHC showed that BAP1 protein expression in the corresponding tumor tissues was negative (shown in **E**, **F**, **G**, **H**, IHC, ×400).

The sequence analysis of the *BAP1* gene from 12 OMM patients revealed missense mutations in four patients (Chr3: 52407995 C>G, p.S113T; Chr3: 52409864 C>A, p.W5C; Chr3: 52408565 T>A, p.E55V; Chr3: 52402325 C>T, p.R718Q) and this included identifying mutations in both the tumor and the blood samples of one of the patients (Table 1 and Figure 1).

**Table 1:** The summary of mutations and nuclear protein expression of BAP1 in 12 OMM patients

No.	Gender	Age	Site	Mutation of tumor sample (hg38)	Mutation of blood sample	nBAP1 expression
1	Female	57	Hard palate	Chr3: 52407995 C>G, p.S113T	Chr3: 52407994 C>G, p. S113T	-
2	Female	46	Hard palate	WT	WT	+
3	Female	78	Maxillary gum& palate	WT	WT	-
4	Male	54	Maxillary gum& palate	Chr3: 52409864 C>A, p.W5C	WT	-
5	Male	71	Mandibular gum	WT	WT	+
6	Male	75	Hard palate	WT	WT	+
7	Female	40	Maxillary gum	WT	WT	-
8	Female	69	Hard palate	Chr3: 52408565 T>A, p.E55V	WT	-
9	Male	48	Mandibular gum	WT	WT	+
10	Female	23	Maxillary gum	WT	WT	+
11	Male	41	Maxillary gum	Chr3: 52402325 C>T, p.R718Q.	WT	-
12	Female	37	Hard palate	WT	WT	+

Abbreviation: nBAP1 expression, nuclear BAP1 expression; WT, wild type.

Of the four mutations, three mutations (Chr3: 52407995 C>G, p.S113T; Chr3: 52409864 C>A, p.W5C; Chr3: 52408565 T>A, p.E55V) were located in the UCH (ubiquitin COOH-terminal hydrolase) domain (amino acids 1-240), whereas the fourth mutation (Chr3: 52402325 C>T, p.R718Q.) was localized to the BRCA1-interaction domain (596-729). Previously, the amino acid sequence from 717 to 722 had been reported as a nuclear localization signal (NLS) [21].

### Clinicopathological data of patients

The age range of the cohort of 62 patients that were investigated in this study was between 25 to 80 years old at diagnosis (mean: 55.4 years). The most common sites of OMM were the hard palate (31/62) and upper gingiva (20/62) (Table 2). The median follow-up period was 35.5 months with a range from 6 months to 9 years. The 3- and 5-year overall survival (OS) times were 46.8% and 25.0%, respectively. Five patients (8.1%) died during the follow-up. Forty patients demonstrated lymphogenous metastasis, whereas, 25 patients had haematogenous metastasis. The histopathological parameters are listed in Table 3.

**Table 2:** Clinical features of 62 patients with primary oral mucosal melanoma

Variable	No. of case (%)
**Age (years)**	
Mean (SD)	55.4 (13.2)
Range	25-80
**Gender n (%)**	
Male	34 (54.8)
Female	28 (45.2)
**Site n (%)**	
Hard plate	31(50.0)
Soft palate	2 (3.2)
Maxillary gum	20 (32.3)
Mandible gum	6 (9.7)
Lip	2 (3.2)
Buccal	3 (4.8)
**Clinical stage n (%)**	
III	14 (22.6)
IVa	41 (66.1)
IVb	4 (6.5)
IVc	3 (4.8)
**Lymphogeneous metastasis**	
Yes	40(64.5)
No	22(35.5)
**Haematogeneous metastasis**	
Yes	25(40.3)
No	37(59.7)

* The lesions of 2 patients involved both hard palate and maxillary gum.

**Table 3:** The prognosis value of nuclear BAP1 expression in OMM patients

Prognostic factors	No. of case	OS (%)		Univariate	Multivariate	HR(95%CI)
	(%)	3-year	5-year	P (log-rank)	P	
**nBAP1 expression**				<0.001 (21.308)	0.022	2.210(1.122-4.352)
Negative	27 (43.5)	18.5	7.4			
Positive	35 (56.5)	68.6	39.0			
**Cell type**				<0.001 (20.272)	0.031	2.579(1.088-6.114)
Epithelioid	32 (51.6)	25.0	9.4			
Non-epithelioid	30 (48.4)	70.0	42.0			
**Ulceration**				0.218 (1.517)	-	-
Absent	15 (24.2)	53.3	40.3			
Present	47 (75.8)	44.7	20.2			
**Mitotic rate**				0.022 (5.234)	0.578	0.991(0.960-1.023)
<1/HPF	34 (54.8)	55.9	34.4			
≥1/HPF	28 (45.2)	35.7	14.3			
**Pigment**				0.100 (2.708)	0.635	1.084(0.776-1.515)
Absent or weak	36 (58.1)	38.9	19.4			
Strong	26 (41.9)	57.7	33.3			
**Necrosis**				0.016 (5.762)	0.078	0.745(0.537-1.033)
Absent	26 (41.9)	65.4	42.3			
Present	36 (58.1)	33.3	12.2			
**Vascular invasion**				0.057 (3.618)	-	-
Absent	58 (93.5)	53.4	26.8			
Present	4 (6.5)	0.0	0.0			

Abbreviations: nBAP1 expression, nuclear BAP1 expression; HR, hazard ratio; 95% CI, 95% confidence interval.

### BAP1 expression is an independent predictor of OS in OMM

**Figure 2:**
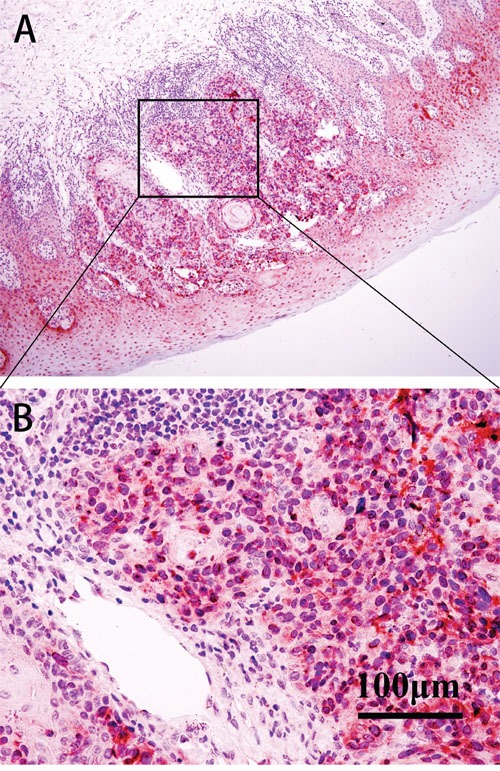
IHC of BAP1 in patient samples The expression of BAP1 protein was predominantly in the nuclei and plasma of tumor cells. **A**. BAP1 in the nuclei of epithelial cells and tumor cells (IHC, ×100). **B**. BAP1 in the nuclei of tumor cells (IHC, ×400).

**Figure 3:**
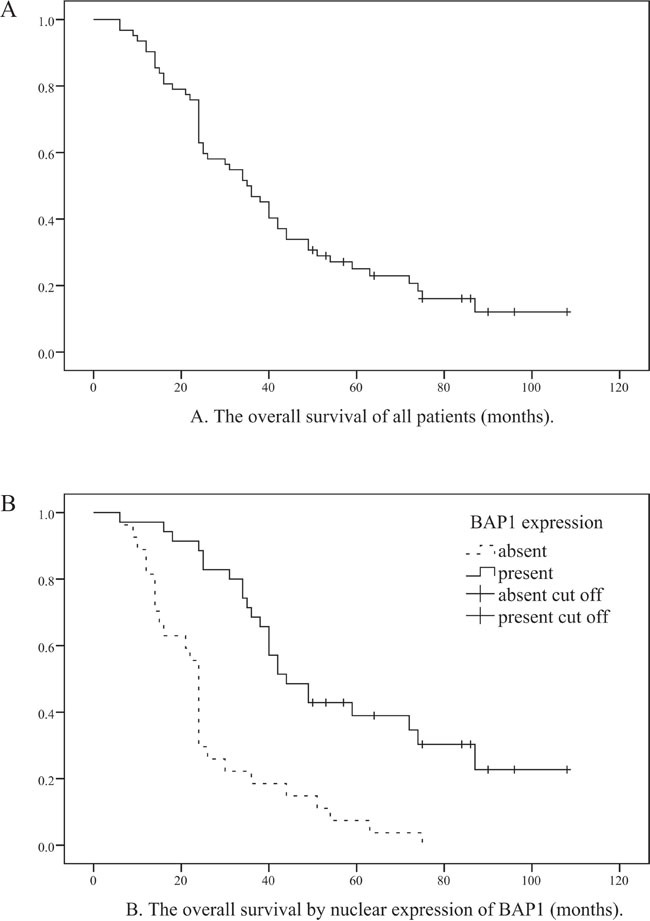
Relationship between overall survival of all OMM patients and nuclear BAP1 protein expression **A**. Kaplan-Meier analysis of 62 OMM patients. **B**. The log-rank test showing that patients with negative nuclear BAP1 expression have poor OS (log-rank=21.308, *P*<001).

Although BAP1 was detected predominantly in the nuclei of tumor cells (Figure 2), weak to moderate expression was also observed in the cytoplasm. Loss of nuclear BAP1 expression was observed in 27 cases and their 3- and 5-year OS rates were 18.5% and 7.4%, respectively. On the other hand the 3- and 5- year OS rates for OMM patients that showed nuclear BAP1 expression were 68.6% and 39.0%, respectively. The loss of nuclear BAP1 expression was associated with poor OS in OMM patients (*P*<0.001, Log-rank=21.308; Figure 3) and the presence of epithelioid cell type (*P*=0.002). In addition, multivariate analysis identified BAP1 as an independent prognostic factor in which cell type, mitotic rate, pigmentation and necrosis were included (*P*=0.022, HR=2.210; Table 3).

### Loss of BAP1 expression is associated with distant metastasis

Based on univariate analysis, we found that the loss of nuclear BAP1 expression was associated with distant metastasis in OMM patients (*P*=0.034, OR=3.125). However, it was not an independent predictor in multivariate analysis in which cell type and tumor infiltrating lymphocytes (TIL) were included (*P*=0.355, OR=1.761). The absence of TIL was an independent predictor of distant metastasis (*P*=0.040, OR=9.616; Table 4).

**Table 4:** Logistic regression analysis of the metastasis risk

Prognostic factors	Univariate	P	Multivariate	P
	OR (95% CI)		OR (95% CI)	
**Loss of nBAP1 expression**	3.125 (1.088 - 8.979)	0.034	1.761 (0.531 - 5.838)	0.355
**Epithelioid cell type**	2.055 (1.188 - 3.557)	0.010	2.835 (0.838 - 9.591)	0.094
**Absence of TIL**	13.000 (1.574-107.354)	0.017	9.616 (1.111-83.268)	0.040
**High mitotic rate**	2.769 (0.972 - 7.888)	0.057	-	-

Abbreviations: nBAP1 expression, nuclear BAP1 expression; OR, odds ratio; 95% CI, 95% confidence interval.

## DISCUSSION

The etiology, prognosis, histopathology and genetic alterations of OMM are different from its cutaneous counterparts and largely unknown [3–5, 22–24]. Melanomas from different mucosal sites have distinctly different features [25]. Nearly 15-30% of mucosal melanomas in the head and neck region harbor activating mutations in BRAF and KIT [26]. In our previous studies, we showed that only 7% tumors harbored *KIT* mutations and 3.5% harbored *BRAF* mutations, whereas, the classic *BRAF V600E* mutation was not detected in OMM and no mutation was found in *NRAS* and *GNAQ/GNA11* [27]. In addition, Miao and colleagues did not find any *TERT* mutation in OMM [28].

Recent studies revealed that *BAP1* mutations are closely associated with the onset and prognosis of melanoma and other malignancies [8]. *BAP1* mutations were detected in 32.5% of uveal melanoma and 2.4% of cutaneous melanoma according to the TCGA database. In this study, we detected *BAP1* mutations in 4 out of 12 patients and the rate of mutation was higher than other classic mutations in OMM. The present study took advantage of the large patient population and the higher incidence of the disease. However, the amount of samples that were submitted for Sanger sequencing were relatively small. One of the reasons for this was the rarity of OMM. Also, in many cases, formalin-fixed and paraffin-embedded samples were not reliable for Sanger sequencing. Therefore, further studies with a larger number of patients with next generation sequencing are necessary to confirm our findings.

OMM is a rare disease with a higher incidence in Asia than in the western countries [29]. We selected 62 OMM patients that were treated at one single large cancer center from 2007 to 2012. Although, the treatment modality of OMM has not been established as yet, the patients included in our study were treated similarly and hence any bias due to variable treatment was controlled. Our data suggested that the loss of nuclear BAP1 expression was associated with poor OS, distant metastasis as well as with the epithelioid cell type, which is a strong histopathological prognostic factor in OMM patients.

The BAP1 protein is a member of the ubiquitin C-terminal hydrolase (UCH) subfamily of deubiquitylating enzymes (DUBs) with ubiquitin carboxyl hydrolase activity and two nuclear localization signal (NLS) motifs. For tumor suppression, both the deubiquitinating activity and nuclear localization are necessary [9]. BAP1 showed predominantly nuclear localization in melanoma cells with wild type *BAP1* [30]. *BAP1* mutation and its correlation with the outcome of malignancies have been widely reported. Loss of nuclear BAP1 protein was associated with aggressive metastatic phenotype and poor prognosis in uveal melanoma [13, 30, 31]. The loss of BAP1 expression, although not frequent in cutaneous melanoma, was independently associated with poor DFS and MSS [14]. Also, the cutaneous melanomas showed functional inactivation (by mutation or epigenetic mechanisms) of *BAP1* [12, 13]. In the present study, loss of nuclear BAP1 was associated with mutations in the BAP1 exons in four of the twelve patient samples analyzed and was an independent prognostic factor that predicted poor OS. Therefore, results of our study concur with previous findings in uveal melanoma and cutaneous melanoma that, suggested that loss of nuclear BAP1 protein expression correlated with an aggressive subtype with poor survival in OMM patients.

In previous studies regarding the histopathological prognostic factors of OMM, the most significant prognostic factor was the cell type [6]. The cell type was also a strong prognostic factor in uveal melanomas [32]. Epithelioid cell type is an aggressive subtype of OMM with poor prognosis and a high risk of distant metastasis [6]. Mutation in *BAP1* has been correlated with the cell type of melanoma in many studies [13, 30, 31]. Loss of BAP1 was also associated with the epithelioid histological type of melanocytic neoplasms including uveal melanoma and epithelioid atypical Spitz tumor [12, 33] and the presence of epithelioid cells in uveal melanoma [31]. The results of this study showed that the epithelioid cell type was associated with the loss of nuclear BAP1 expression in OMM. Recent reports have associated the loss of BAP1 expression with reduced melanocytic differentiation in melanomas [17, 34]. Therefore, BAP1 appears to function in the uveal melanocyte lineage primarily as a regulator of differentiation, with cells deficient for BAP1 exhibiting stem-like features [17, 34]. Meanwhile, histone deacetylase (HDAC) inhibitors induced differentiation and prolonged dormancy of micro-metastatic disease in uveal melanoma [17]. Also, the loss of *BAP1* could regulate class I HDAC expression and affect the sensitivity of tumor cells to HDAC inhibitors [16]. Our study demonstrates that understanding the functional mechanism of BAP1 in OMM could have important therapeutic implications in the future.

Although, BAP1 is associated with metastasis in uveal melanoma, its functional mechanism was unknown [11]. In this study, we found that loss of nuclear BAP1 expression was associated with distant metastasis. However, it was not an independent predictor when TIL and cell type were included in the multivariate model, whereas, the absence of TIL was an independent predictor of distant metastasis. TIL is a well established prognostic factor in cutaneous melanoma and an independent predictor of the distant metastasis in OMM [6, 35]. The presence of TIL is part of the host resistance mechanism to melanoma cells.

In conclusion, we detected four mis-sense *BAP1* mutations in the twelve OMM patient samples that were sequenced. The loss of nuclear BAP1 expression was associated with OS and distant metastasis and the univariate analysis identified BAP1 as an independent prognostic factor of OMM.

## MATERIALS AND METHODS

### DNA extraction and sanger sequencing

The biopsy specimens obtained from the patients were confirmed to be OMM at the Department of Oral Pathology, Shanghai Ninth People's Hospital. The tumor samples were matched with their blood samples that were used as control. All the samples were stored at −80°C prior to DNA extraction. The DNA was extracted using the DNeasy Blood & Tissue KIT (Qiagen, Shanghai, China) according to the manufacturer's protocol and genomic DNA was amplified by PCR. Each 20μl PCR reaction contained 10 ng DNA, Thermo-start ReadyMix PCR Master Mix (ThermoScientific) and 0.2μM forward and reverse PCR primers, respectively. The PCR conditions were: one cycle of 95°C for 15min, 35cycles of 95°C for 30 sec, 58 °C for 30 sec and 72 °C for 1 min, followed by one final extension cycle of 72 °C for 4 min. The sequences of primers are shown in Supplementary Table 1. PCR products were purified using SAP/Exo I (Applied Biosystems, CA, USA). Bidirectional DNA sequencing was performed in the ABI 3730XL instrument (Applied Biosystems) by Shanghai Sangon Biotechnology Co., Ltd. (Shanghai, China). We then analyzed the *BAP1* DNA sequences from 12 primary OMM patients. DNA sequences were analyzed against hg38 reference sequences using Seqman program of Lasergene 7.1 software (DNASTAR Inc., Wisconsin, USA).

### Clinical details of patients

The present study was performed in accordance with the Helsinki declaration and was approved by the Ethics Committee of Shanghai Ninth People's Hospital. We enrolled 62 primary OMM patients (34 Males and 28 Females) that were treated at the Shanghai Ninth People's Hospital between April 2007 to April 2012 for IHC assessment and survival analysis. The inclusion criteria and treatment modalities were as described in our previous study [6]. Briefly, the inclusion criteria for the patients in the study were: (1) The primary lesion site was oral mucosal and (2) The complete clinical and pathological data of the patients was available including gender, age, primary lesion site, clinical stage and therapy modalities. The exclusion criteria for patients were: (1) The patients had melanoma in other sites or other malignancies; (2) The patients were above 80 years of age when diagnosed; (3) The patients had been treated by immunotherapy, chemotherapy or radiotherapy before the pathological diagnosis or (4) The patients refused to receive treatment. IHC assessment of Melan-A, S-100 and HMB45 was used to confirm the diagnosis in case of doubt.

A radical resection or cryotherapy was performed for primary lesions. Cryotherapy was initially suggested to treat superficial lesions. Prophylactic, functional, or radical neck dissection was performed for different patients [18, 19]. Post-operative chemotherapy was performed every 3 weeks for 2 cycles in CN0 patients and for 4 cycles in CLN positive patients. Patients with distant metastasis at diagnosis were treated by chemotherapy as palliative care. The histopathological prognostic factors, including cell type, ulceration, mitotic rate, pigmentation, necrosis, vascular invasion and tumor infiltrating lymphocyte (TIL) were evaluated as described in our previous study [6].

Survival was measured from the date of pathologic diagnosis. Follow-up was carried out every 2-4 months during the first year, every 4-6 months in the second year and every 6 months thereafter. Computed tomography (CT) scans of the craniomaxillofacial-neck region and the lungs were performed every 6 months. Positron emission computed tomography (PET-CT) was performed if distant metastasis was suspected. Patients that were still alive by December 2015 or had died during follow up were censored from the analysis.

### Staining of BAP1 immunohistochemistry

The primary tumor tissues obtained from 74 primary OMM patients (62 patients for IHC and survival analysis and 12 patients for Sanger sequencing, as described in previous sections) were fixed in formalin, embedded in paraffin and 4μm thick sections were cut. When cytological details were obscured due to heavy pigmentation, the sections were bleached to remove melanin.

For IHC staining of BAP1, the tissue sections were deparaffinized with xylene and rehydrated with decreasing concentrations of ethanol. The sections were then treated with 3% peroxide to block endogenous peroxidase activity and heat-treated for unmasking. The sections were incubated at 4°C overnight with rabbit polyclonal antibody to BAP1 (C-4, sc-28383, Santa Cruz) at a dilution of 1: 100. IHC was performed on a DAKO Real Envision Detection System (DAKO, Copenhagen, DK) with AEC peroxidase substrate (Vector Laboratories) according to the manufacturer's instructions. As control for non-specific antibody binding, the primary antibody was substituted with PBS. The nuclei of the keratinocytes served as positive internal controls.

BAP1 protein expression was evaluated by two independent investigators who were blinded to the clinical data of the patients. Any disagreements were resolved by discussion. The nuclear BAP1 expression was scored as positive or negative. Only nuclear staining of BAP1 was considered to be positive.

### Statistical analysis

All statistical analysis was performed by PASW Statistics 18.0.0. The nuclear BAP1 expression was evaluated against the overall survival (OS) of the patients using the Kaplan-Meier curves and performing the log-rank tests (n=62). Cox proportional hazards model analysis was performed after controlling for other factors to determine the influence of BAP1 on OS (n=62). Histopathological parameters including cell type, ulceration, mitotic rate, pigmentation, necrosis and vascular invasion were analyzed in the Kaplan-Meier curves and significant prognostic parameters were included for multivariate analysis. The assessment of these histopathological parameters was as described in a previous study [6]. Logistical regression was applied to evaluate the odds ratios for distant metastases (n=62). The absence of TIL, cell type and mitotic rate was analyzed and the significant parameters were included in the multivariate analysis. Two sided statistical tests were performed and a p<0.05 was considered significant.

## SUPPLEMENTARY MATERIALS TABLE



## References

[1] Lourenco SV, A MS, Sotto MN, Bologna SB, Giacomo TB, Buim ME, Coutinho-Camillo CM, Silva SD, Landman G, Soares FA, Simonsen Nico MM (2009). Primary oral mucosal melanoma: a series of 35 new cases from South America. The American Journal of dermatopathology.

[2] Temam S, Mamelle G, Marandas P, Wibault P, Avril MF, Janot F, Julieron M, Schwaab G, Luboinski B (2005). Postoperative radiotherapy for primary mucosal melanoma of the head and neck. Cancer.

[3] Gavriel H, McArthur G, Sizeland A, Henderson M (2011). Review: mucosal melanoma of the head and neck. Melanoma research.

[4] Rapini RP, Golitz LE, Greer RO, Krekorian EA, Poulson T (1985). Primary malignant melanoma of the oral cavity. A review of 177 cases. Cancer.

[5] Meleti M, Leemans CR, Mooi WJ, Vescovi P, van der Waal I (2007). Oral malignant melanoma: a review of the literature. Oral oncology.

[6] Song H, Wu Y, Ren G, Guo W, Wang L (2015). Prognostic factors of oral mucosal melanoma: histopathological analysis in a retrospective cohort of 82 cases. Histopathology.

[7] Song H, Jing G, Wang L, Guo W, Ren G (2015). Periodic acid-Schiff-positive loops and networks as a prognostic factor in oral mucosal melanoma. Melanoma research.

[8] Carbone M, Yang H, Pass HI, Krausz T, Testa JR, Gaudino G (2013). BAP1 and cancer. Nature reviews Cancer.

[9] Murali R, Wiesner T, Scolyer RA (2013). Tumours associated with BAP1 mutations. Pathology.

[10] Jensen DE, Proctor M, Marquis ST, Gardner HP, Ha SI, Chodosh LA, Ishov AM, Tommerup N, Vissing H, Sekido Y, Minna J, Borodovsky A, Schultz DC (1998). BAP1: a novel ubiquitin hydrolase which binds to the BRCA1 RING finger and enhances BRCA1-mediated cell growth suppression. Oncogene.

[11] Harbour JW, Onken MD, Roberson ED, Duan S, Cao L, Worley LA, Council ML, Matatall KA, Helms C, Bowcock AM (2010). Frequent mutation of BAP1 in metastasizing uveal melanomas. Science.

[12] Wiesner T, Murali R, Fried I, Cerroni L, Busam K, Kutzner H, Bastian BC (2012). A distinct subset of atypical Spitz tumors is characterized by BRAF mutation and loss of BAP1 expression. The American journal of surgical pathology.

[13] Koopmans AE, Verdijk RM, Brouwer RW, van den Bosch TP, van den Berg MM, Vaarwater J, Kockx CE, Paridaens D, Naus NC, Nellist M, van Ijcken WF, Kilic E, de Klein A (2014). Clinical significance of immunohistochemistry for detection of BAP1 mutations in uveal melanoma. Modern pathology.

[14] Murali R, Wilmott JS, Jakrot V, Al-Ahmadie HA, Wiesner T, McCarthy SW, Thompson JF, Scolyer RA (2013). BAP1 expression in cutaneous melanoma: a pilot study. Pathology.

[15] Pfoh R, Lacdao IK, Saridakis V (2015). Deubiquitinases and the new therapeutic opportunities offered to cancer. Endocrine-related cancer.

[16] Sacco JJ, Kenyani J, Butt Z, Carter R, Chew HY, Cheeseman LP, Darling S, Denny M, Urbe S, Clague MJ, Coulson JM (2015). Loss of the deubiquitylase BAP1 alters class I histone deacetylase expression and sensitivity of mesothelioma cells to HDAC inhibitors. Oncotarget.

[17] Landreville S, Agapova OA, Matatall KA, Kneass ZT, Onken MD, Lee RS, Bowcock AM, Harbour JW (2012). Histone deacetylase inhibitors induce growth arrest and differentiation in uveal melanoma. Clinical cancer research.

[18] Wang X, Wu HM, Ren GX, Tang J, Guo W (2012). Primary oral mucosal melanoma: advocate a wait-and-see policy in the clinically N0 patient. Journal of oral and maxillofacial surgery.

[19] Wu Y, Zhong Y, Li C, Song H, Guo W, Ren G (2014). Neck dissection for oral mucosal melanoma: caution of nodular lesion. Oral oncology.

[20] Yang X, Ren GX, Zhang CP, Zhou GY, Hu YJ, Yang WJ, Guo W, Li J, Zhong LP (2010). Neck dissection and post-operative chemotherapy with dimethyl triazeno imidazole carboxamide and cisplatin protocol are useful for oral mucosal melanoma. BMC cancer.

[21] Misaghi S, Ottosen S, Izrael-Tomasevic A, Arnott D, Lamkanfi M, Lee J, Liu J, O’Rourke K, Dixit VM, Wilson AC (2009). Association of C-terminal ubiquitin hydrolase BRCA1-associated protein 1 with cell cycle regulator host cell factor 1. Molecular and cellular biology.

[22] Guevara-Canales JO, Gutierrez-Morales MM, Sacsaquispe-Contreras SJ, Sanchez-Lihon J, Morales-Vadillo R (2012). Malignant melanoma of the oral cavity. Review of the literature and experience in a Peruvian Population. Medicina oral, patologia oral y cirugia bucal.

[23] Lourenco SV, Fernandes JD, Hsieh R, Coutinho-Camillo CM, Bologna S, Sangueza M, Nico MM (2014). Head and neck mucosal melanoma: a review. The American Journal of dermatopathology.

[24] Barker BF, Carpenter WM, Daniels TE, Kahn MA, Leider AS, Lozada-Nur F, Lynch DP, Melrose R, Merrell P, Morton T, Peters E, Regezi JA, Richards SD (1997). Oral mucosal melanomas: the WESTOP Banff workshop proceedings. Western Society of Teachers of Oral Pathology. Oral surgery, oral medicine, oral pathology, oral radiology, and endodontics.

[25] Prasad ML, Busam KJ, Patel SG, Hoshaw-Woodard S, Shah JP, Huvos AG (2003). Clinicopathologic differences in malignant melanoma arising in oral squamous and sinonasal respiratory mucosa of the upper aerodigestive tract. Archives of pathology & laboratory medicine.

[26] Curtin JA, Busam K, Pinkel D, Bastian BC (2006). Somatic activation of KIT in distinct subtypes of melanoma. Journal of clinical oncology.

[27] Lyu J, Wu Y, Li C, Wang R, Song H, Ren G, Guo W (2015). Mutation scanning of BRAF, NRAS, KIT, and GNAQ/GNA11 in oral mucosal melanoma: a study of 57 cases. Journal of oral pathology & medicine.

[28] Miao Y, Wang R, Ju H, Ren G, Guo W, Lyu J (2015). TERT promoter mutation is absent in oral mucosal melanoma. Oral oncology.

[29] Takagi M, Ishikawa G, Mori W (1974). Primary malignant melanoma of the oral cavity in Japan. With special reference to mucosal melanosis. Cancer.

[30] Shah AA, Bourne TD, Murali R (2013). BAP1 protein loss by immunohistochemistry: a potentially useful tool for prognostic prediction in patients with uveal melanoma. Pathology.

[31] Kalirai H, Dodson A, Faqir S, Damato BE, Coupland SE (2014). Lack of BAP1 protein expression in uveal melanoma is associated with increased metastatic risk and has utility in routine prognostic testing. British journal of cancer.

[32] McLean IW, Foster WD, Zimmerman LE, Gamel JW (1983). Modifications of Callender’s classification of uveal melanoma at the Armed Forces Institute of Pathology. American journal of ophthalmology.

[33] Wiesner T, Obenauf AC, Murali R, Fried I, Griewank KG, Ulz P, Windpassinger C, Wackernagel W, Loy S, Wolf I, Viale A, Lash AE, Pirun M (2011). Germline mutations in BAP1 predispose to melanocytic tumors. Nature genetics.

[34] Matatall KA, Agapova OA, Onken MD, Worley LA, Bowcock AM, Harbour JW (2013). BAP1 deficiency causes loss of melanocytic cell identity in uveal melanoma. BMC cancer.

[35] Clark WH, Elder DE, Dt Guerry, Braitman LE, Trock BJ, Schultz D, Synnestvedt M, Halpern AC (1989). Model predicting survival in stage I melanoma based on tumor progression. Journal of the National Cancer Institute.

