# Supplementary material for: Loss of nuclear BAP1 expression is associated with poor prognosis in oral mucosal melanoma
Source: Oncotarget. 2017 Mar 14;8(17):29080–90. doi: 10.18632/oncotarget.16175 (PMC5438714; doi:10.18632/oncotarget.16175)
Supplement: Supplementary file 1 [file oncotarget-08-29080-s001.pdf]

Loss of nuclear BAP1 expression is associated with poor prognosis in oral mucosal melanoma

SUPPLEMENTARY TABLE

Supplementary Table 1: Sequencing primers for evaluation of *BAP1* mutations by Sanger sequencing

Region	Primer Sequence (5' -> 3')	PCR product Size (bp)
**Exon 1**	F: ACCGAAATCTTCCACGAGCA<br>R: CTGGACATGGCGCTGAGG	202
**Exon 2-3**	F: GGAGAGCGACCCAGGTGAGG<br>R: GGGTTCCTGGCACTGTCTTCC	398
**Exon 4**	F: CACTTCAGACACAGTGTTGGGAAG<br>R: CGTTCTGCCAGAGGATTTCTGTAG	430
**Exon 5**	F: GAGTTGTCCAGATATGACTGACCTG<br>R: GGAAACTCTCCTCCCTCCCTAG	450
**Exon 6-7**	F: GTTCGTCTGTGTTCCTTCCGATTC<br>R: CTGGTCGGGCAATATGGTGTAG	569
**Exon 8**	F: CTACACCATATTGCCCGACCAG<br>R: CTAAGCCTGATCTTGCCAGATTCAC	333
**Exon 9**	F: GTGCCTGGCATGTATGGCTAGTC<br>R: GTGGTTAGCTGAAGCCCAGATC	410
**Exon 10**	F: CTGTGAGTGAATGGGTAGAGCCAAG<br>R: CTGCTCTCCCTCTACCTTCTGAC	406
**Exon 11**	F: CTTAGAGCTTGCTGACTCCCATTG<br>R: CATATCAGGCAGAGGAACCTAGCAAC	478
**Exon 12**	F: CTATCCAGTGTAAGTGGGTGGCAG<br>R: CTCCGCAGGTGCTCAACATTATC	426
**Exon 13**	F: GTTGCTTGGACCAAGTATAAGGAG<br>R: CTCTGGGAAGAGAGGTCACAAG	730
**Exon 14**	F: GACAGGTGGGCCTTGGACTG<br>R: CAACCCAGAAAGTCTTCTGGCAC	426
**Exon 15-16**	F: GTGCCAGAAGACTTTCTGGGTTG<br>R: CAGGGCATTCCAGTTAAGACAG	550
**Exon 17**	F: CTGTCTTAACTGGAATGCCCTG<br>R: CTGGTTCCTCCCATTCCCAG	441
